# Effect of soil and community factors on the yield and medicinal quality of *Artemisia argyi* growth at different altitudes of the Funiu mountain

**DOI:** 10.3389/fpls.2024.1430758

**Published:** 2024-10-29

**Authors:** Di Yang, Xingqiao Liu, Xinao Xu, Tongfei Niu, Xiaolei Ma, Guozhan Fu, Chengwei Song, Xiaogai Hou

**Affiliations:** College of Agriculture/Tree Peony, Henan University of Science and Technology, Luoyang, Henan, China

**Keywords:** *A. argyi*, wild population, altitude, medicinal quality, soil, mineral elements

## Abstract

Altitude and ecological factors significantly influence plant growth and the accumulation of secondary metabolites. However, current research on the impact of altitude and ecological factors on the yield and medicinal quality of *Artemisia argyi (A. argyi)* is limited. This study established sampling sites in wild populations of *A. argyi* across seven altitude ranges on Funiu Mountain. We quantified the yield, output rate of moxa, and key medicinal ingredients. Additionally, we analyzed the response of yield and medicinal quality of wild *A. argyi* populations to various ecological factors at different altitudes. The results showed that wild populations of *A. argyi* exhibited higher yields and medicinal quality at altitudes below 500 m. Yield was positively correlated with higher soil total nitrogen (TN) content and lower soil total phosphorus (TP) content, while the improvements in medicinal quality were positively associated with higher population density and lower contents of both soil TN and TP. The variation in soil C/N, C/P, and N/P ratios across different altitudes was substantial, affecting soil mineralization and subsequently influencing the absorption of mineral elements by *A. argyi*. Notably, the phosphorus content in leaves and stems was negatively correlated with yield and medicinal quality, respectively. In contrast, the accumulation of nitrogen, phosphorus, and potassium in leaves was positively correlated with yield. The differences in the primary medicinal ingredients between the leaves and stems of *A. argyi* were maximum at altitudes below 500 m. The contents of neochlorogenic acid and cryptochlorogenic acid in both leaves and stems showed a significant positive correlation. In the principal component analysis, the primary medicinal ingredients from the leaves contributed more significantly to the overall quality than those from stems. These results suggest that *A. argyi* is best suited for cultivation at altitudes below 500 m. Population density and the soil’s TN and TP contents play a crucial role in determining the yield and medicinal quality of *A. argyi*. Futhermore, the medicinal quality of *A. argyi* is more indicative of the main medicinal ingredients found in the leaves, while the stems also serve as a key organ for accumulating flavonoids and phenolic acids.

## 1 Introduction

Plant-based Chinese medicinal materials have predominantly been sourced from the wild, with their growth being influenced by various biological and abiotic factors. The synthesis and accumulation of secondary metabolites represent a crucial defense strategy for adapting to stress, while also serving as the foundation for their efficacy. Altitude acts as a crucial geographical factor, leading to variations in local environmental conditions, which subsequently affect the characteristics and functionalities of plant species. Altitude heterogeneity considerably impacts the yield and medicinal quality of plants used for medicinal purposes. For instance, the roots of *Angelica sinensis* exhibit an increase in both yield and ferulic acid content as altitude increases (Wang et al., 2013a, b). In addition, the growth and quality formation of medicinal plants are influenced by various other factors, including slope aspect, slope gradient, soil physical and chemical properties, as well as the diversity of associated flora (Tashi and Sushila, 2022). The natural habitats of *Artemisia argyi (A. argyi)* occur in arid slopes, roadside areas, riverbanks, and other locations that are susceptible to drought, intense sunlight, temperature fluctuations, nutrient deficiencies, weed competition, and various other factors. Elucidating the adaptive mechanisms employed by *A. argyi* to cope with such habitat stress is of immense significance for achieving high-yield and high-quality cultivation of this species.

The mineral elements and organic compounds in medicinal plants predominantly exist as complexes, playing a pivotal role in determining efficacy and influencing the yield of medicinal materials. Nitrogen (N), phosphorus (P), and potassium (K) are essential elements present in significant quantities and are extensively involved in the growth, development, and synthesis of numerous secondary metabolites. The dry matter and artemisinin yield of *Artemisia annua* correlated positively with the combined application of N and P (Aftab et al., 2016). A scientifically combined application of N, P, and K can effectively promote the growth of *Panax ginseng* and *Chrysanthemum morifolium*, alter the rhizosphere soil microbial community, increase its adaptability to environmental conditions, and thus improve both quality and yield (Sun et al., 2022; Fang et al., 2023). In studies of *A. argyi*, the contents of phenolic acids and flavonoids significantly increased under low N stress, while high N stress improved plant growth and photosynthesis, leading to improvement quality of volatile oil (Wang et al., 2023a). The contents of phenolic acids and flavonoids showed a negative correlation with the amount of P applied (Wang et al., 2023b). Currently, large-scale cultivation of *A. argyi* has been carried out in over 10 provinces in China (Ma et al., 2021a), resulting in substantial variations in planting habitats. However, the relationship between the yield of *A. argyi*, its medicinal quality, and mineral elements across different habitats has poorly documented.

The active ingredients in different organs of medicinal plants are closely related, yet their accumulation patterns can vary significantly. For example, the primary metabolite of the roots, stems, leaves and flowers of *Salvia miltiorrhiza* is phenolic acid; however, there are differences in content, type and synthesis pathways (Tong et al., 2022). Similarly, the flavonoid content and antioxidant capacity of the petals and receptacles of *Chrysanthemum morifolium* also show variation (Lu et al., 2020). In American ginseng, the ginsenoside content is the highest in the root, followed by the stem and then the leaf (Gong et al., 2023). The entire *A. argyi* plant possesses medicinal properties, with its primary medicinal part being the leaves, which are rich in volatile oils, flavonoids, phenolic acids, and other bioactive constituents. This composition renders the leaves suitable for direct medicinal use as well as for moxa. At present, research on the medicinal value of different organs of *A. argyi* is limited and not comprehensive. As *A. argyi* leaves are dry leaves taken without flowering in summer (Chinese Pharmacopoeia Commission, 2020), and the harvested parts include leaves and stems, it is necessary to carry out research on the medicinal value of leaves and stems and analyze the relationship between them.

Funiu Mountain, located in Henan Province, China, is one of the authentic production regions for *A. argyi*. This area shows significant variations in altitude and encompasses diverse wild habitats for *A. argyi*. To analyze the impact of altitude and ecological factors on yield and medicinal quality in wild populations of *A. argyi*, as well as to identify key ecological factors, we conducted comprehensive assessments that included measurements of soil chemical properties, community characteristics, individual yield, output rate of moxa, and the contents of major medicinal ingredients and mineral elements in different organs. This study aims to provide scientific insights into the ecological adaptability of *A. argyi* and promote the high-quality cultivation practices that enhance yields.

## 2 Materials and methods

### 2.1 Overview of the study area

The study area is located in the southern foothills of Funiu Mountain in Henan Province. Altitudes in this region range from 72.2 to 2212.5 m. The average annual rainfall varies from 703.6 to 1173.4 mm, while the average annual temperature is between 14.4 and 15.7 °C. The frost-free period spans 220 to 245 days, and the total annual sunshine duration ranges from 1897.9 to 2120.9 h. The community types in this section include forest, scrub, shrub-grass, and grassland. *A. argyi* is found in patches within the shrub or tussock communities.

### 2.2 Methods

#### 2.2.1 Sample plot settings and sample collection

**Figure 1:**
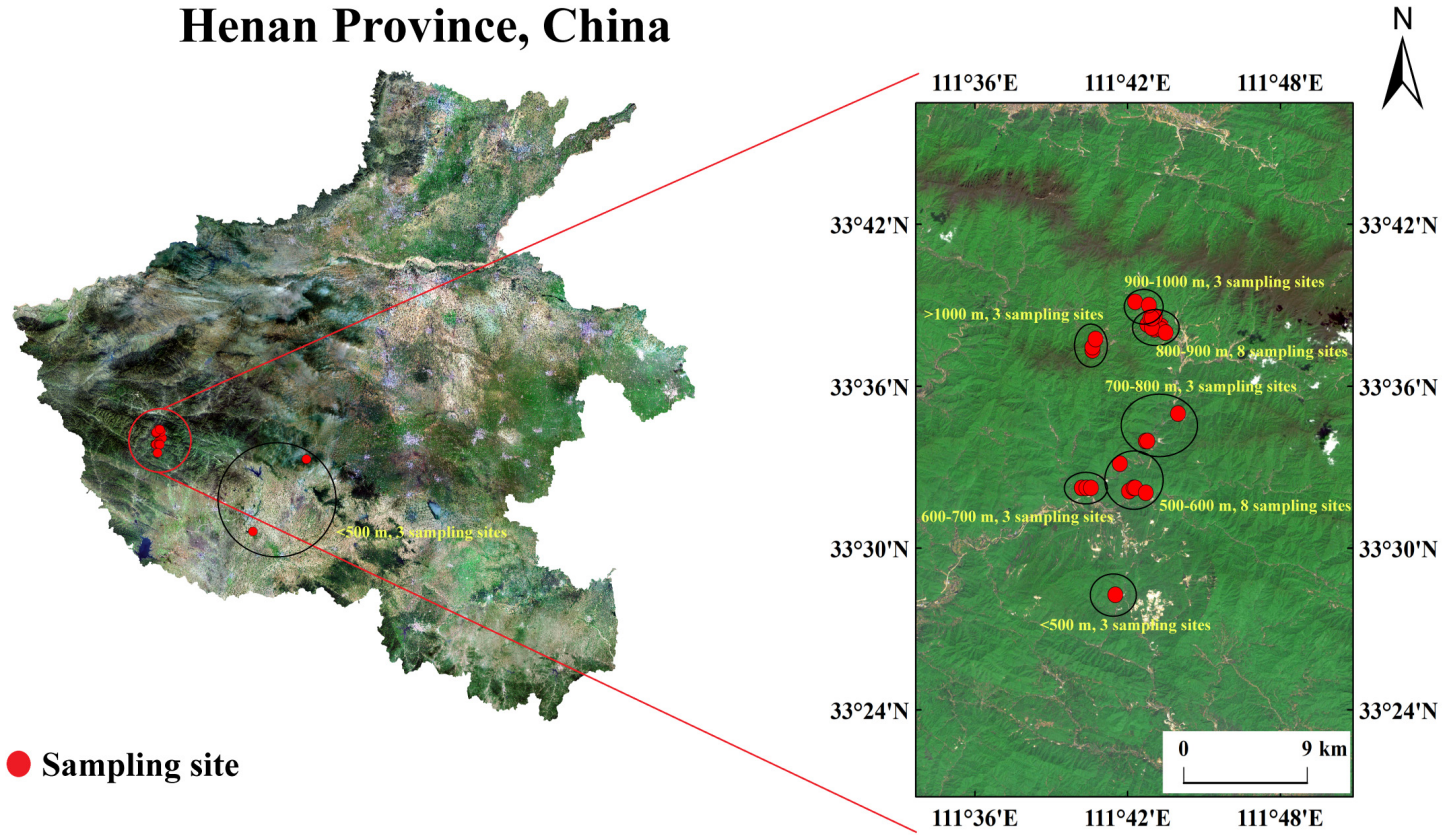
Distribution of sampling sites on Funiu Mountain.

The shrub-grass and grass communities associated with *A. argyi* were selected for investigation in the study area. Based on the actual distribution of *A. argyi*, we identified 31 wild populations, each larger than 25 m^2^, as our study subjects across seven altitude ranges. To maintain consistency during the growth period, we conducted a harvest in each population around June 3, 2022. Following a 60-day growth period after the harvest (from late July to early August), we carried out uniform collection. The seven altitude ranges were categorized as follows: <500 m, 500-600 m, 600-700 m, 700-800 m, 800-900 m, 900-1000 m, and >1000 m, respectively. The number of populations within each altitude range was: 3, 8, 3, 3, 8, 3, and 3. For specific population distribution information, please refer to **Figure 1**. During sampling, we selected non-flowering plants with stable phenotypic traits as standard. Within each population, three replicates were established, each containing twenty individuals (a total of sixty individuals). The average values from these replicates represented the yield and medicinal quality of each population. The mean values from all populations within each altitude range represented the yield and medicinal quality for that range. Additionally, based on the size of each population area, we established three to five herb plots measuring two meters by two meters to assess community factors. We recorded and measured plant species using ecological counting methods, including family, genus, species, coverage rate, height. Furthermore, we collected soil samples using a five-point sampling method at depths ranging from zero to twenty centimeters within each population for chemical analysis.

#### 2.2.2 Calculation of community characteristic factors

In this study, the importance value (IV) of shrub and herb species was used to assess their relative significance, indicating species dominance within each quadrat. Species richness index (D), Shannon-Wiener index (H), Simpson index (H′), and Pielou’s evenness index (J) were employed to evaluate community species diversity. The slope aspect index (TRP) was utilized to assess the heliotropism of the community habitats. Population density was measured to quantify the number of individuals per unit area within the wild population. The plant species’ ecotypes in the quadrat were classified as heliophytes, shade-demanding plants, or shade-enduring plants. The calculation formula was as follows (Guo et al., 2021; Chen et al., 2023):

1. IV=(relative coverage+relative height)/2 ;
2. TRP={1−cos[(π/180)(aspect−30)]}/2 ;
3. D=S ;
4. $\text{H}=-\sum_{i=1}^{S}P_{i}lnP_{i}$ ;
5. H'=1−∑i=1sPi2 ;
6. J=H/lnD ;

In these formulae, TRP is the slope aspect index, and the closer the value is to 1, the sunnier and drier the habitat is. aspect is slope aspect. S is the number of community species. *P_i_* is the important value of species *i*.

#### 2.2.3 Soil chemical properties

The soil samples were air-dried, ground, and sieved. Soil organic matter (SOM) and soil organic carbon (SOC) contents were determined using the external heating method of potassium dichromate oxidation. Total nitrogen (TN) and total phosphorus (TP) contents were determined using a high-resolution digital colorimeter auto analyzer 3 (AA3, SEAL Company, Germany). The readily available potassium (AK) contents in the soil was determined by flame spectrophotometry (Model 410 Flame Photometer, Sherwood, UK), while the pH value was assessed in a 1:2.5 soil-water ratio suspension using a pH meter (PHSJ-4F, Leici, China) (Bao, 2013; Shaaban et al., 2023).

#### 2.2.4 Growth traits

Plant height is measured from the base to the top of the *A. argyi*. Stem diameter refers to the middle section of the main stem of *A. argyi*. Distance of five leaves represents the length from the mature leaf at the top of *A. argyi* to the fifth leaf. Leaf length and leaf width are defined as the length and width of three mature leaves located in the middle of the main stem, respectively.

#### 2.2.5 Individual yield and main medicinal ingredients

Individual yield involves collecting all the leaves from a single *A. argyi* plant and determining their dry weight after they have been in the shade drying. Output rate of moxa is the ratio of the moxa weight to the weight of the *A. argyi* leaf sample. The determination of total volatile oil was performed following the method outlined General Rule 2204, titled “Determination Method of Volatile Oil” as described in the Chinese Pharmacopoeia (Fourth Part) (2020 edition). The contents of eucalyptus oleoresin and borneol were determined using the collected volatile oil as in conjunction with gas chromatography and mass spectrometry (Agilent 5977C, GC-MS, USA). The concentrations of eight phenolic acids and flavonoids were determined using a high-performance liquid chromatograph (Agilent 1260 Infinity II, HPLC, USA), which included six phenolic acids (neochlorogenic acid, chlorogenic acid, cryptochlorogenic acid, isochlorogenic acid B, isochlorogenic acid A, and isochlorogenic acid C) as well as two flavonoids (jaceosidin and eupatilin) (Ma et al., 2021b).

#### 2.2.6 Content of mineral elements in different organs

Mineral element contents of leaves and stems were assessed as follows: N and P contents were determined using a high-resolution digital colorimeter auto analyzer 3 (AA3, SEAL Company, Germany), while K contents were determined by flame spectrophotometry (Model 410 Flame Photometer, Sherwood, UK) (Bao, 2013; Zhang et al., 2024). The contents of N, P, and K in leaves are referred to as leaf nitrogen content (LNC), leaf phosphorus content (LPC), and leaf potassium content (LKC), respectively. Their corresponding accumulation amounts are recorded as leaf nitrogen accumulation (LNA), leaf phosphorus accumulation (LPA), and leaf potassium accumulation (LKA). Similarly, N, P, and K contents in stems are referred to as stem nitrogen content (SNC), stem phosphorus content (SPC), and stem potassium content (SKC).

### 2.3 Statistical analysis

The data was collated using Excel 2019, while variance analysis, principal component analysis, comprehensive evaluation, RDA redundancy analysis, correlation analysis and drawing were performed using SPSS 17.0, Canoco 5.0, GraphPad Prism 9.5 and Origin 2021. The species cumulative curve was established using R 4.3.2, and the sampling sites distribution map was drawn using ArcGIS 10.2.

## 3 Results

### 3.1 Growth traits, individual yield and medicinal quality of *A. argyi* at different altitudes

**Figure 2:**
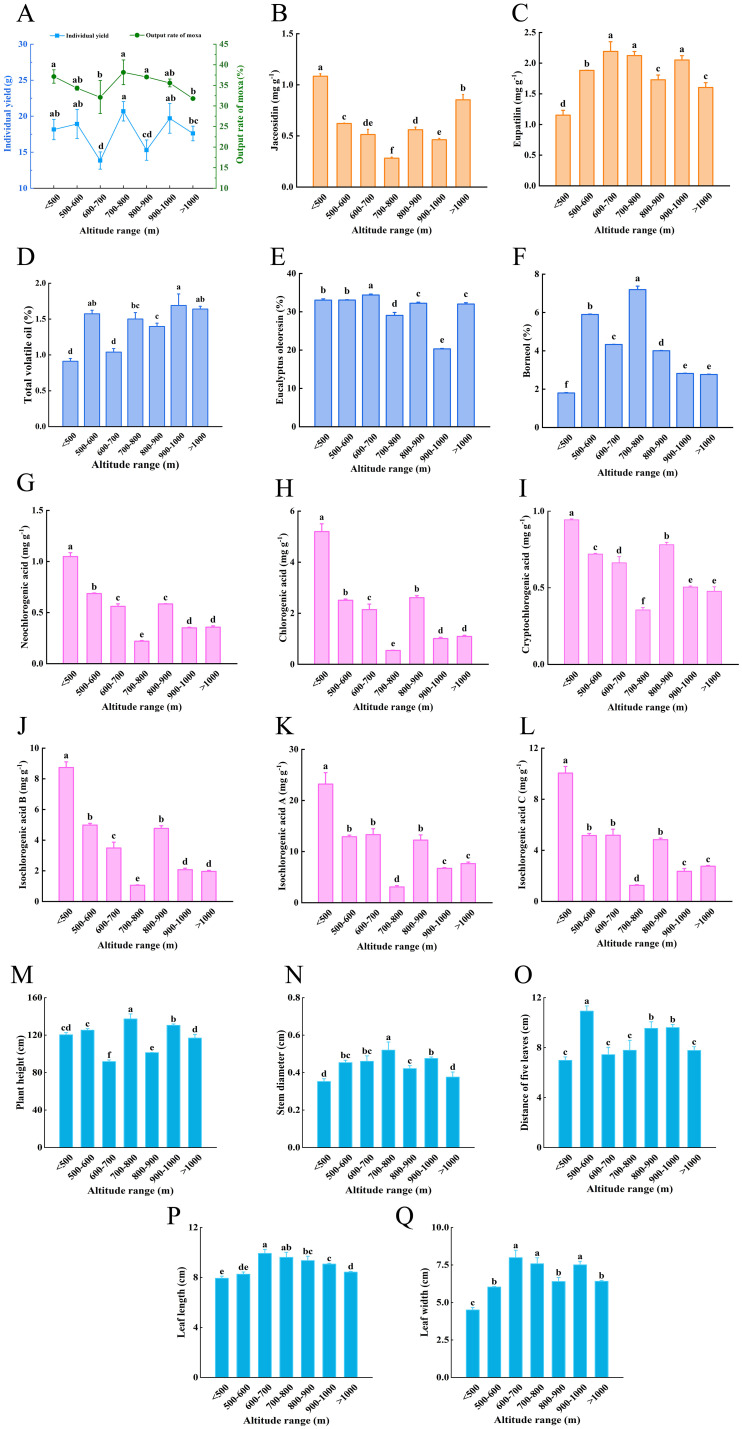
Individual yield, leaf quality and growth traits of wild population of *A*. *argyi* at different altitudes **(A)**, Individual yield and output rate of moxa **(B)**, Content of jaceosidin **(C)**, Content of eupatilin **(D)**, Content of total volatile oil **(E)**, Content of eucalyptus oleoresin **(F)**, Content of borneol **(G)**, Content of neochlorogenic acid **(H)**, Content of chlorogenic acid **(I)**, Content of cryptochlorogenic acid **(J)**, Content of isochlorogenic acid B **(K)**, Content of isochlorogenic acid A **(L)**, Content of isochlorogenic acid C **(M)**, Plant height **(N)**, Stem diameter **(O)**, Distance of five leaves **(P)**, Leaf length **(Q)**, Leaf width. Different lowercase letters above bars indicated significant difference (*P*<0.05).

The variation in individual yield and medicinal quality with altitude was inconsistent and varied significantly. The individual yield, output rate of moxa, and borneol content peaked at an altitude range of 700-800 m, reaching values of 20.68 g, 38.17%, and 7.19%, respectively (**Figures 2A, F**). The maximum total volatile oil content of 1.69% was achieved within the altitude range of 900-1000 m (**Figure 2D**). Meanwhile, the eucalyptus oleoresin content reached its peak of 34.36% in the 600-700 m altitude range (**Figure 2E**). The variation trends of neochlorogenic acid, chlorogenic acid, cryptochlorogenic acid, isochlorogenic acid B, isochlorogenic acid A, isochlorogenic acid C, and jaceosidin followed a similar pattern. Below 800 m, the contents gradually decreased with increasing altitude, reaching a minimum between 700-800 m, which was significantly lower than values observed at other altitude ranges. Above 800 m, with the exception of jaceosidin, which increased above 1000 m, the contents of the other 6 components showed fluctuations, initially increasing before ultimate decreasing, with their maximum values found below 500 m (**Figures 2B, G–L**). The content of eupatilin exhibited a pattern of increase-decrease-increase-decrease with rising altitude, achieving a maximum value in the 600-700 m range and a minimum below 500 m (**Figure 2C**). The growth traits of *A. argyi* across different altitude ranges showed significant differences (**Figures 2M–Q**). Plant height, stem diameter, and individual yield exhibited a consistent trend, peaking at an altitude range of 700-800 m, significantly higher than at other altitudes. The distance of five leaves was greatest in the 500-600 m altitude range, while leaf length and width were relatively larger in the 600-700 m and 700-800 m ranges.

### 3.2 Comprehensive evaluation of medicinal quality of *A. argyi* at different altitudes

Three principal components (PC1, PC2, PC3) were extracted from the principal component analysis of the medicinal quality of *A. argyi* at different altitudes, revealing a cumulative interpretation rate of 89.512%. This rate effectively reflects overall information related to medicinal quality. The variance contribution rate of PC1 was 67.733%, where the absolute values of the eigenvectors for the contents of neochlorogenic acid, chlorogenic acid, isochlorogenic acid B, isochlorogenic acid C, cryptochlorogenic acid, isochlorogenic acid A, jaceosidin, eupatilin, total volatile oil, eucalyptus oleoresin and borneol were larger. The variance contribution rate of PC2 was 11.683%, with the absolute values of the eigenvectors for eucalyptus oleoresin and borneol were higher. This suggests that the first two principal components indicate how *A. argyi* adjusts its active ingredients in response to changes in altitude. The variance contribution rate of PC3 was 10.097%, where the absolute value of the eigenvectors for output rate of moxa was larger, reflecting the response of output rate of moxa to altitude changes (**Table 1**).

**Table 1:** Principal component analysis of medicinal quality of *A. argyi* leaves at different altitude intervals.

Index	PC1	PC2	PC3
Output rate of moxa	0.046	0.264	0.932
Total volatile oil	-0.741	-0.369	0.09
Eucalyptus oleoresin	0.530	0.501	-0.492
Borneol	-0.563	0.752	0.009
Neochlorogenic acid	0.976	0.097	0.042
Chlorogenic acid	0.985	0.087	0.102
Isochlorogenic acid B	0.972	0.092	0.13
Isochlorogenic acid C	0.988	0.086	0.009
Jasceosidin	0.828	-0.438	-0.158
Eupatilin	-0.778	0.372	-0.188
Cryptochlorogenic acid	0.938	0.119	0.04
Isochlorogenic acid A	0.981	0.059	-0.042
Initial eigenvalue	8.128	1.402	1.212
Contribution rate/%	67.733	11.683	10.097
Accumulative contribution rate/%	67.733	79.416	89.512

PC1, PC2 and PC3 represent principal component 1, principal component 2 and principal component 3, respectively.

**Figure 3:**
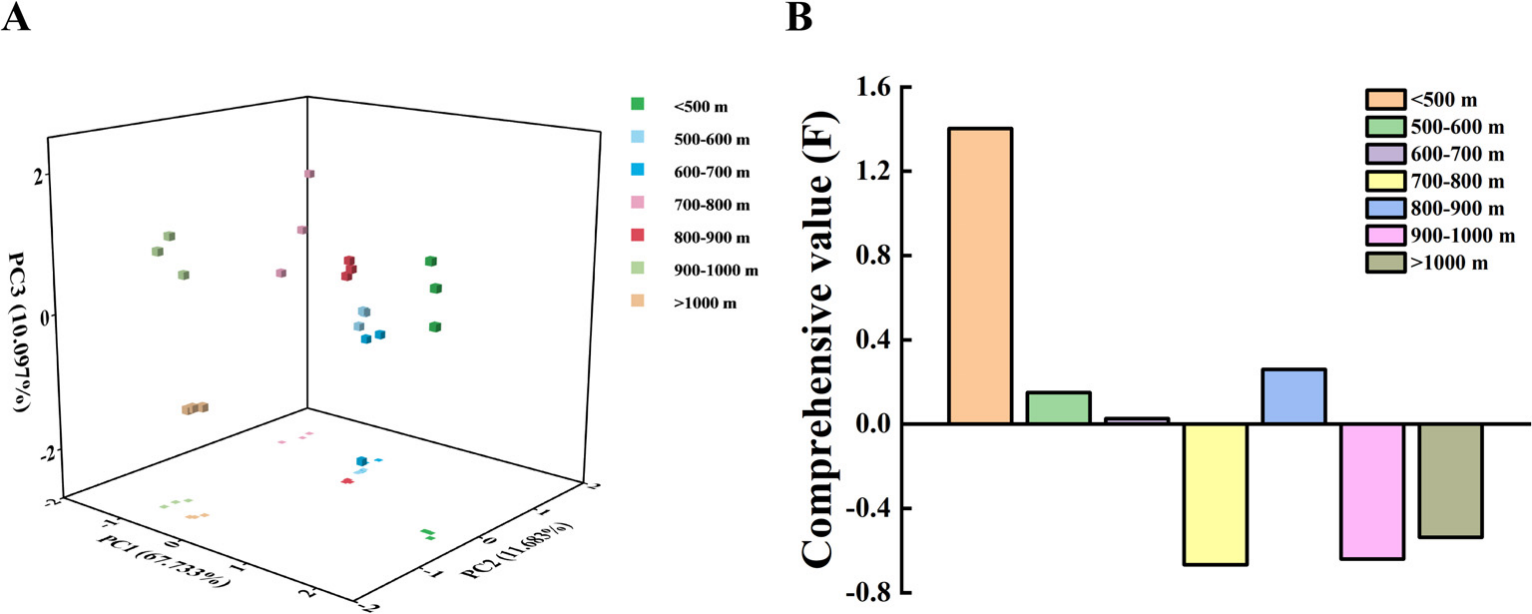
PCA diagram and comprehensive value of medicinal quality of *A*. *argyi* leaves at different altitudes **(A)**, PCA diagram **(B)**, Comprehensive value.

According to the results of the PCA diagram, the medicinal quality at altitudes of 500-600 m, 600-700 m, and 800-900 m was similar, while the other four altitude intervals were distinctively grouped into separate categories, showing obvious differences in medicinal quality (**Figure 3A**). Comprehensive values were calculated and ranked based on the membership function values. The altitude intervals, arranged from highest to lowest comprehensive value of medicinal quality of *A. argyi*, were <500 m, 800-900 m, 500-600 m, 600-700 m, >1000 m, 900-1000 m, and 700-800 m. The highest medicinal quality of *A. argyi* was observed at altitudes below 500 m (**Figure 3B**).

### 3.3 Community characteristics and soil chemical properties of wild populations of *A. argyi* at different altitudes

The slope of communities at different altitudes ranged from 1.40° to 49.05°, with habitats generally displaying steeper slopes, except in the range of 700-800 m. The TRP of the communities at different altitudes varied from 0.09 to 0.94. Habitats below 700 m were drier and sunnier compared to those above 700 m. Population density varied greatly ranging from 8.5 to 24.92 plants m^-2^ (**Table 2**).

**Table 2:** Basic community information of wild populations of *A. argyi* at different altitudes.

Altitude (m)	Slope (°)	TRP	Population density (plants m^-2^)
<500	31.33	0.79	24.92
500-600	12.78	0.78	12.5
600-700	49.05	0.94	15.38
700-800	1.40	0.09	8.5
800-900	19.95	0.47	18.75
900-1000	26.35	0.55	9.92
>1000	16.67	0.38	18.5

**Figure 4:**
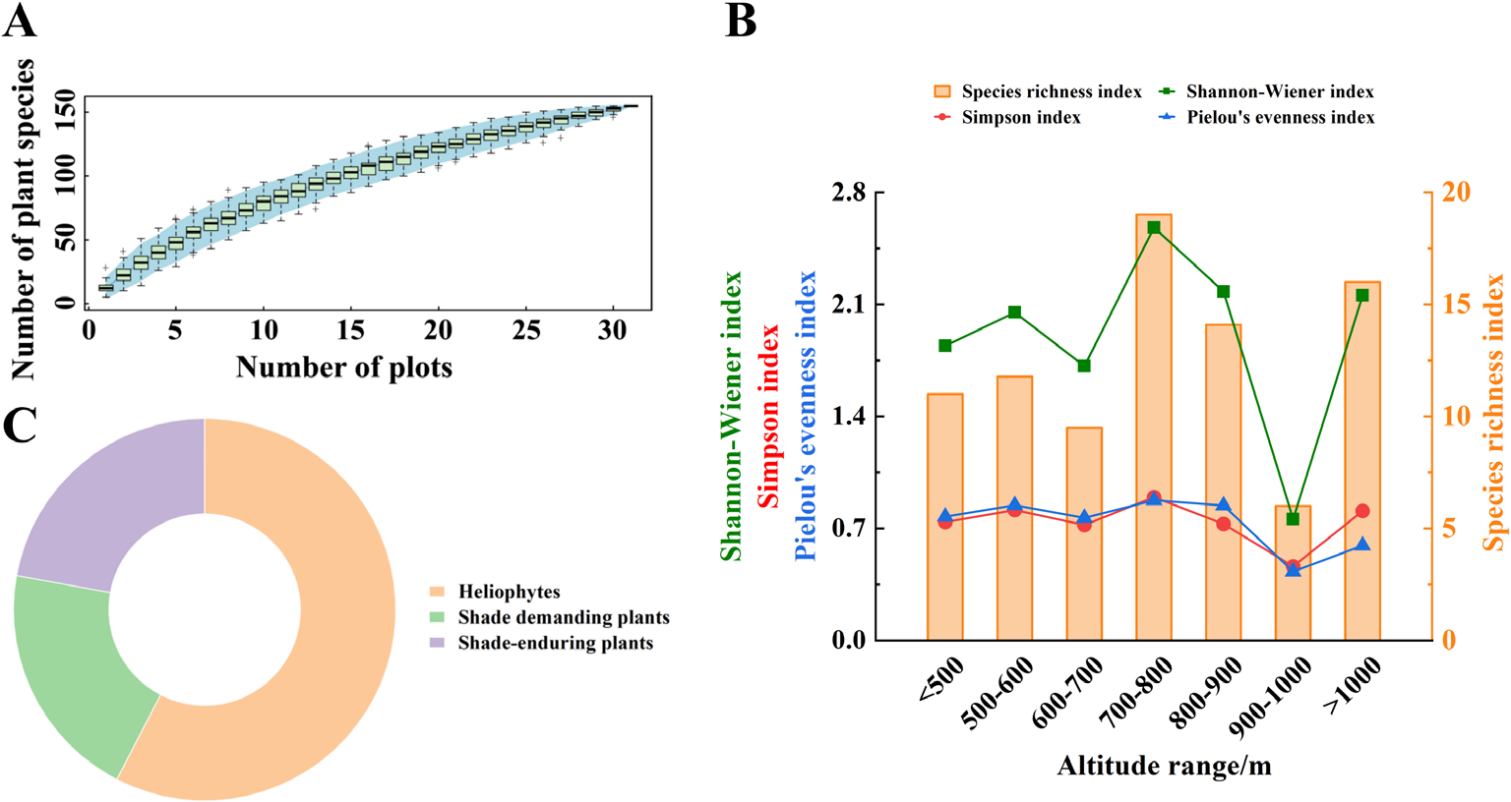
Community species structure of wild populations of *A*. *argyi* at different altitudes **(A)**, Species accumulation curve of the community **(B)**, Species diversity characteristics of the community **(C)**, Ecotype classification of community species.

The plant richness within the quadrats initially increased with the number of quadrats but gradually slows down, eventually reaching an asymptotic line. This trend indicates that most species present in the area have been documented, demonstrating the effectiveness of quadrat set up (**Figure 4A**). A total of 155 plant species from 50 families and 110 genera were investigated. As altitude increases, species richness, the Shannon-Wiener index, the Simpson index, and Pielou’s evenness index consistently exhibit similar patterns within the *A. argyi* communities, peaking at 700-800 m and reaching a minimum at 900-1000 m (**Figure 4B**). Based on the ecological adaptability of plants to light intensity, the ecotypes of *A. argyi* communities at different altitudes consist of 58% heliophytes, 20% shade-demanding plants, and 22% shade-enduring plants, with heliophytes being dominant group (**Figure 4C**).

**Figure 5:**
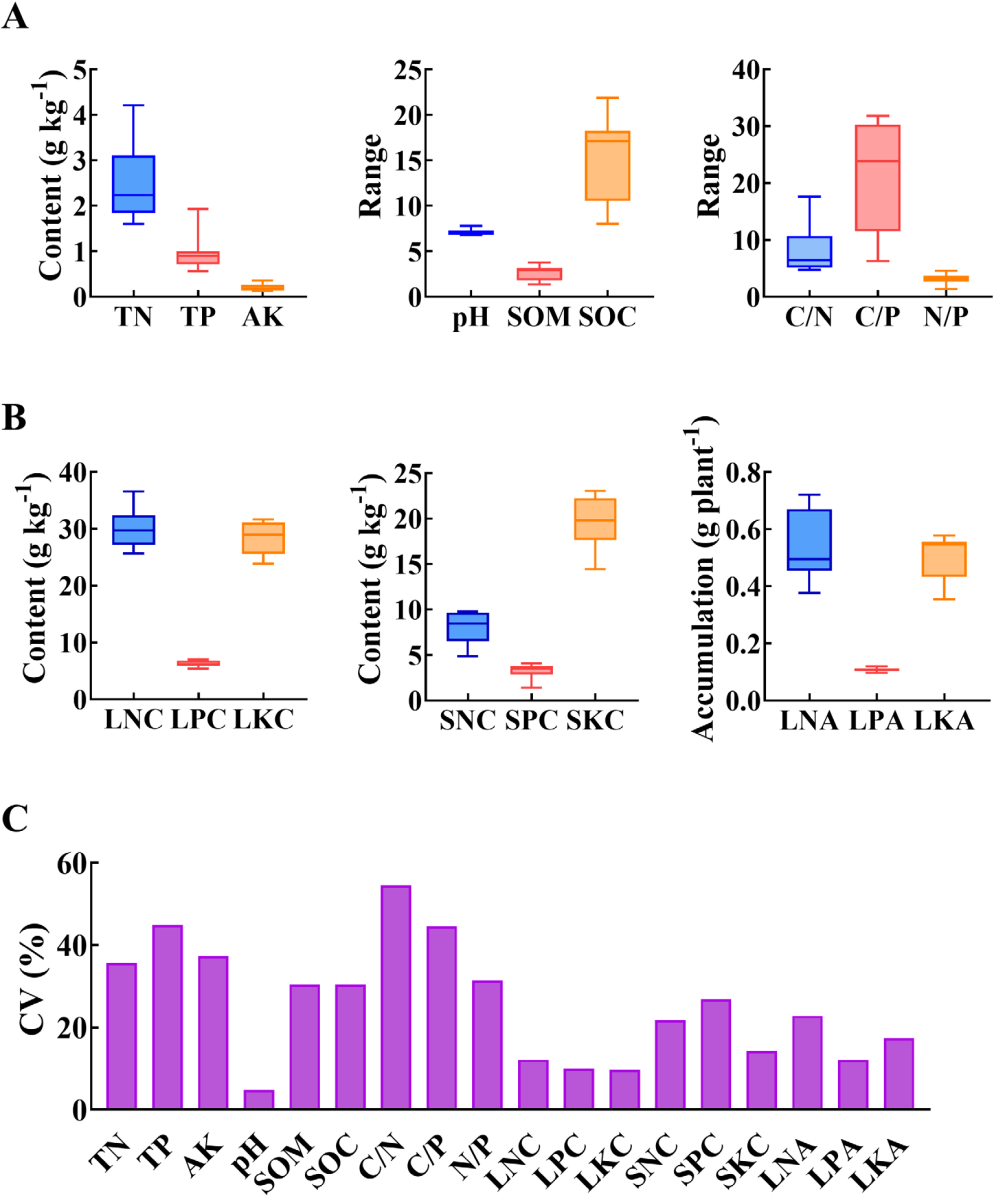
Characteristics of soil chemical properties and mineral elements accumulation of different organs at different altitude. LNC, LPC, LKC indicated the contents of N, P, and K in leaves, respectively. SNC, SPC, SKC indicated the contents of N, P, and K in stems, respectively. LNA, LPA, LKA indicated the accumulation of N, P, and K in leaves, respectively. The unit of SOM is “%”. The unit of SOC is “g kg^-1^”. **(A)**, Soil chemical properties **(B)**, Mineral elements accumulation characteristics in different organs **(C)**, Coefficient of variation (CV) of indexes of soil chemical properties and mineral elements accumulation of different organs.

The soil TN, TP and AK contents of wild populations of *A. argyi* at different altitudes ranged from 1.60 to 4.21 g kg^-1^, 0.56 to 1.93 g kg^-1^, and 0.13 to 0.35 g kg^-1^, respectively. The pH, SOM and SOC contents were 6.82-7.80, 1.38%-3.77% and 8.02-21.86 g kg^-1^, respectively. Soil carbon-nitrogen ratio (C/N), soil carbon-phosphorus ratio (C/P) and soil nitrogen-phosphorus ratio (N/P) were 4.78-17.61, 6.31-31.81 and 1.40-4.60, respectively (**Figure 5A**). Overall, the soil chemical properties exhibited significant variation, with an uneven distribution of nutrients observed across different altitudes. According to Wilding’s classification (Cui et al., 2021) soil property variation based on the coefficient of variation, (coefficient of variation <15%, small variation; 16%-35%, medium variation; >36%, highly variable), soil pH displayed small variation, TN, SOM, SOC, and N/P exhibited medium variation, while TP, AK, C/N, C/P showed high variation (**Figure 5C**). The LNC, LPC and LKC of wild populations of A. argyi at different altitudes ranged from 25.65 to 36.59 g kg-1, 5.39 to 7.01 g kg-1, and 23.84 to 31.65 g kg-1, respectively. The SNC, SPC and SKC for these populations ranged from 4.84 to 9.77 g kg-1,1.40 to 4.07g kg-1, and 14.42 to 23.04 g kg-1, respectively. The LNA, LPA and LKA varied between 0.38 to 0.72 g plant-1,0.09 to 0.12 g plant-1, and 0.35 to 0.58 g plant-1, respectively (**Figure 5B**). The accumulation characteristics of N, P and K in leaves and stems exhibited considerable variability at different altitudes. The coefficient of variation for LKC was relatively was small at 9.72%, while the coefficients of variation for the indices ranged between 10% and 30% (**Figure 5C**).

### 3.4 Correlation analysis

**Figure 6:**
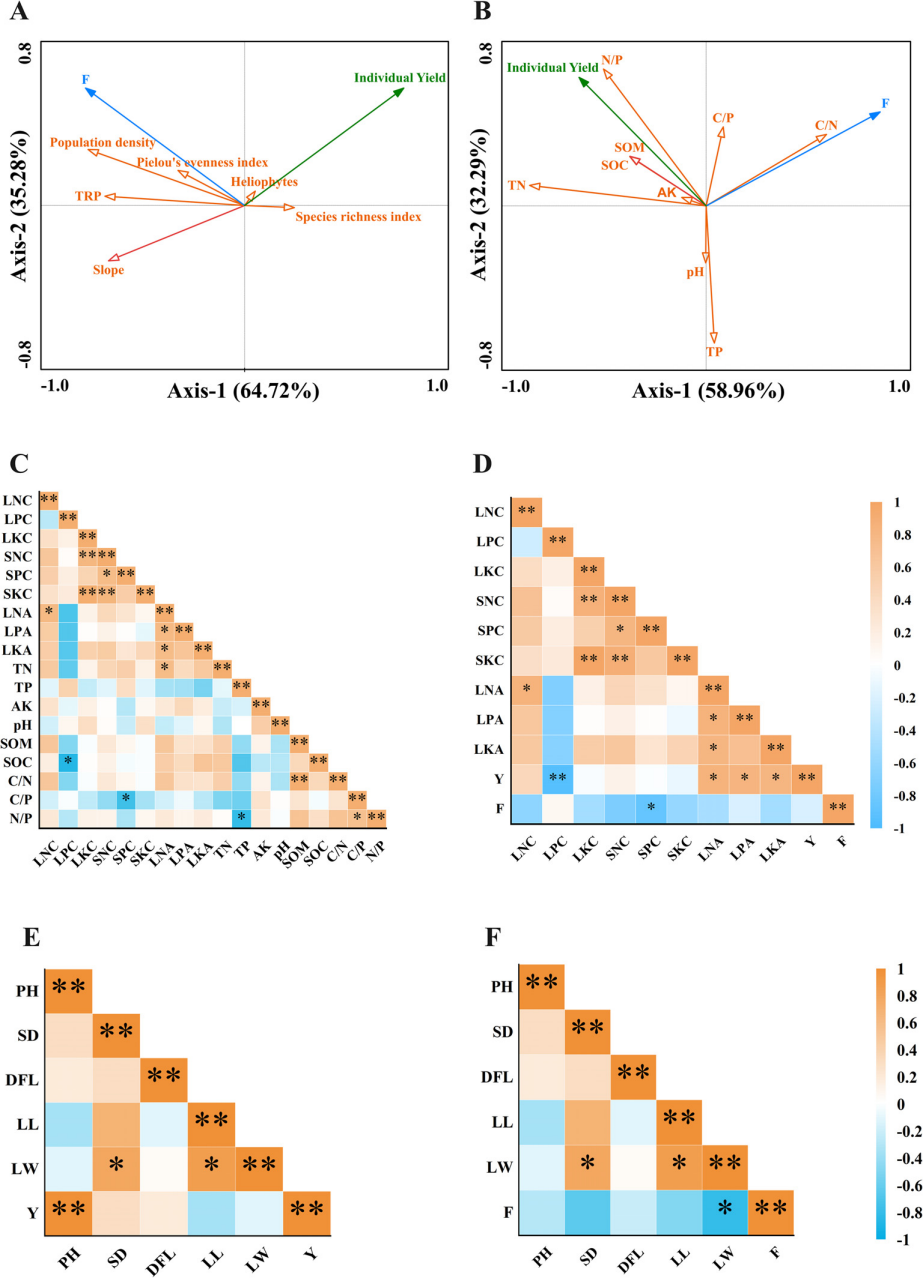
Correlation analysis. LNC, LPC, LKC indicated the contents of N, P, and K in leaves, respectively. SNC, SPC, SKC indicated the contents of N, P, and K in stems, respectively. LNA, LPA, LKA indicated the accumulation of N, P, and K in leaves, respectively. PH, SD, DFL, LL, LW indicated Plant height, Stem diameter, Distance of five leaves, Leaf length, Leaf width. Y indicated the individual yield. F indicated the comprehensive value of medicinal quality of *A*. *argyi* leaves. **(A)**, RDA ordination among community characteristic factors, individual yield and medicinal quality **(B)**, RDA ordination among soil chemical properties, individual yield and medicinal quality **(C)**, Correlation of indexes of soil chemical properties and mineral elements accumulation characteristics of different organs **(D)**, Correlation of mineral elements accumulation characteristics of different organs, individual yield and medicinal quality. **(E)**, Correlation between growth traits and individual yield **(F)**, Correlation between growth traits and medicinal quality. * and ** indicated significant difference at *P*<0.05 and *P*<0.01, respectively.

The comprehensive value (F) of medicinal quality of leaves was used to represent the medicinal quality of *A. argyi*, while RDA ordination was used to analyze the community characteristic factors, soil chemical properties, and their effects on individual yield and medicinal quality. The total explanatory power of community characteristic factors concerning the individual yield and medicinal quality of *A. argyi* was 100%, with 64.72% and 35.28% accounted for by the first and second axes, respectively. The connection between population density, TRP, and slope was represented by relatively long arrows, indicating a greater contribution to individual yield and medicinal quality. Furthermore, these three indexes displayed a negative correlation with individual yield, but a positive correlation with medicinal quality. Notably, population density demonstrated the strongest association with medicinal quality (**Figure 6A**). The total explanatory power of soil chemical factors on the individual yield and medicinal quality of *A. argyi* was 91.25%, with 58.96% and 32.29% explained by the first and second axes, respectively. The arrows representing TN, N/P, C/N and TP were relatively long, indicating their significant contribution to individual yield and medicinal quality. Both TN and N/P showed a positive correlation with individual yield but a negative correlation with medicinal quality. Conversely, C/N was positively correlated with medicinal quality, while TP showed a negative correlation with both individual yield and medicinal quality (**Figure 6B**).

Regarding soil chemical properties, TP showed a negative correlation with N/P (*P*<0.05), while C/P showed a positive correlation with N/P (*P*<0.05). Moreover, was positively correlated (*P*<0.01) with the C/N. In terms of mineral elements in different plant organs, SNC showed significant positive correlations with SPC (*P*<0.05), SKC (*P*<0.01), LKC (*P*<0.01). LNA showed significant positive correlations with LNC (*P*<0.05), LPA(*P*<0.05), LKA (*P*<0.05). LKC showed a positive correlation with SKC (*P*<0.01). Correlations were also observed between soil chemical properties and mineral elements across various organs. Notably, SOC correlated significantly with and LPC, C/P correlated with SPC, TN correlated with LNA (*P* < 0.05) (**Figure 6C**). The accumulation characteristics of mineral elements in different organs affected the yield and medicinal quality of *A. argyi* leaves. LPC was negatively correlated with individual yield (*P*<0.01), whereas LNA, LPA and LKA were positively correlated with individual yield (*P*<0.05). SPC showed a negative correlation with medicinal quality (*P*<0.05) (**Figure 6D**). In terms of growth traits, there is a significant positive correlation between plant height and individual plant yield (*P*<0.01), while leaf width showed a significant negative correlation with the medicinal quality of *A. argyi* leaves (*P*<0.05) (**Figures 6E, F**).

Monte Carlo test was performed for eight community characteristic factors. The importance ranking results of these factors on individual yield and medicinal quality were as follows: Population density>Slope>Heliophytes>Pielou’s evenness index>TRP>Species richness index. The interpretation rate for population density was found to be 43.4%, exhibiting a significant impact on both individual yield and medicinal quality (*P*<0.05), while other factors did not exhibit significant effects (**Table 3**). Likewise, a Monte Carlo test was performed on nine soil chemical factors. The importance ranking results of these factors on individual yield and medicinal quality were as follows: TN>N/P>AK>pH>SOC>TP>SOM>C/N>C/P. TN, N/P and pH demonstrated significant effects on both individual yield and medicinal quality (*P*<0.01), with interpretation rates of 47.0%, 23.5% and 5.6%, respectively. AK, SOC and TP had significant effects on both individual yield and medicinal quality (*P*<0.05), with explanation rates of 6.6%, 4.0% and 3.7%, respectively. The contribution rates of the remaining three factors were relatively low, and their effects did not reach statistical significance (**Table 4**).

**Table 3:** Effects of community characteristic factors on individual yield and medicinal quality.

Indexes	Sequence	Interpretation (%)	*P*
Population density	1	43.4	0.042*
Slope	2	17.1	0.246
Heliophytes	3	12.6	0.264
Pielou’s evenness index	4	16.2	0.148
TRP	5	8.7	0.176
Species richness index	6	2.0	1.000

*indicated significant difference at *P*<0.05.

**Table 4:** Effects of soil chemical properties on individual yield and medicinal quality.

Indexes	Sequence	Interpretation (%)	*P*
TN	1	47.0	0.002**
N/P	2	23.5	0.002**
AK	3	6.6	0.014*
pH	4	5.6	0.008**
SOC	5	4.0	0.034*
TP	6	3.7	0.018*
SOM	7	0.4	0.456
C/N	8	0.4	0.526
C/P	9	<0.1	0.874

* and ** indicated significant difference at *P*<0.05 and *P*<0.01, respectively.

Multiple linear stepwise regression analysis showed no significant regression relationship between individual yield and community characteristic factors. However, a significant multiple linear regression relationship was identified between the medicinal quality of *A. argyi* (*P*<0.05) and population density, with a determination coefficient of 0.648. Individual yield was significantly (*P*<0.01) affected by TN, TP. Medicinal quality was significantly (*P*<0.01) affected by TN, pH, and TP. Both TN and TP emerged as common factors affecting both individual yield and medicinal quality of *A. argyi* (**Table 5**). Integrating the results of RDA ordination, we found population density, along with soil TN and TP contents, as common factors influencing both yield and medicinal quality.

**Table 5:** Stepwise regression analysis among community characteristic factors, soil chemical properties, yield and medicinal quality.

Stepwise regression equation	*R^2^*	Impact factors	*P*
—	—	—	—
F=0.805D_1_-1.589	0.648	Population density	0.029
Y=15.736 + 0.600X_1_-0.424X_3_	0.538	TN,TP	<0.001
F=8.759-0.894X_1_-0.416X_2_-0.366X_3_	0.785	TN,pH,TP	<0.001

Y indicated the individual yield. F indicated the comprehensive value of medicinal quality. D_1_ indicated the population density. X_1_, X_2_, X_3_ indicated the TN, pH and TP, respectively.

### 3.5 Analysis of the main medicinal ingredients of different organs in *A. argyi*

To analyze the main medicinal ingredients in stems of *A. argyi*, we measured the contents of six phenolic acids, including neochlorogenic acid and chlorogenic acid, along with two flavonoids, jaceosidin and eupatilin (**Table 6**). The analysis revealed significant variability in the contents of these phenolic acids and flavonoids at different altitudes. Among them, the coefficients of variation for chlorogenic acid, jaceosidin, and eupatilin were relatively high, with values of 41.52%, 40.23% and 42.50%, respectively. There was a notable disparity between the maximum and minimum values for each index, highlighting the variability in their concentrations.

**Table 6:** Content of main medicinal ingredients in stems at different altitudes.

Indexes	Minimum	Maximum	Mean	Standard deviation	CV (%)
Neochlorogenic acid (mg g-1)	0.09	0.15	0.12	0.02	15.35
Chlorogenic acid (mg g^-1^)	0.10	1.72	0.91	0.38	41.52
Isochlorogenic acid B (mg g^-1^)	2.43	6.97	3.81	1.28	33.58
Isochlorogenic acid C (mg g^-1^)	0.67	1.68	1.04	0.26	25.25
Jaceosidin (mg g^-1^)	0.02	0.13	0.07	0.03	40.23
Eupatilin (mg g^-1^)	0.0006	0.0029	0.0012	0.0005	42.50
Cryptochlorogenic acid (mg g^-1^)	0.09	0.23	0.14	0.04	29.85
Isochlorogenic acid A (mg g^-1^)	0.73	2.1	1.20	0.36	29.65

**Figure 7:**
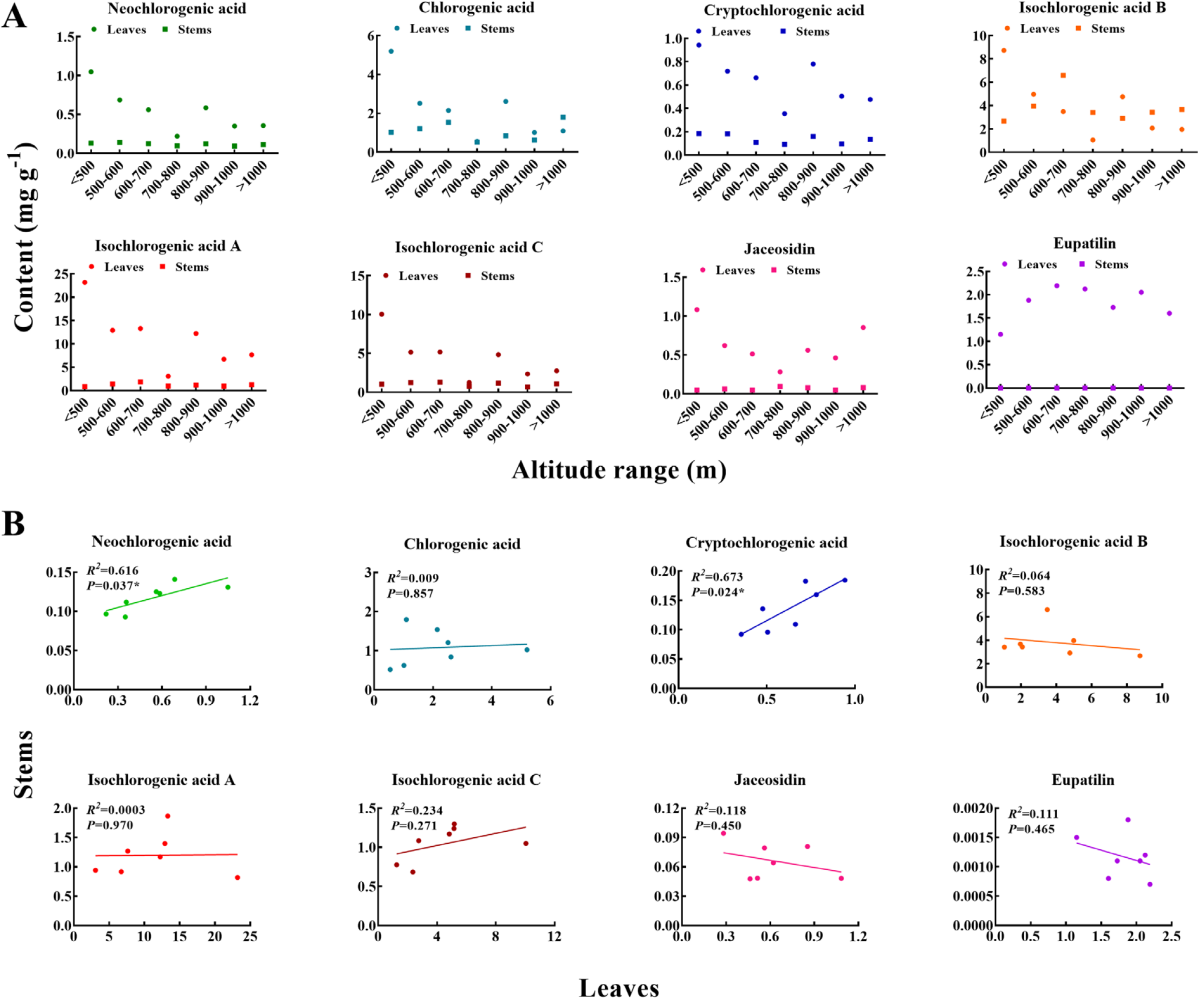
Differences in the contents of main medicinal ingredients and their correlation analysis in different organs at different altitudes **(A)**, Differences in the contents of main medicinal ingredients in different organs at different altitudes **(B)**, Correlation analysis of the contents of main medicinal ingredients in different organs at different altitudes. * indicated significant difference at *P*<0.05.

We compared the contents of eight flavonoids and phenolic acids in both stems and leaves (**Figure 7A**). The levels of neochlorogenic acid, cryptochlorogenic acid, isochlorogenic acid A, isochlorogenic acid C, jaceosidin, and eupatilin were consistently higher in the leaves compared to the stems across all seven altitude ranges. However, chlorogenic acid content exceeded that of the leaves at altitudes above 1000 m, while isochlorogenic acid B was also higher in stems than in leaves at altitudes above 600 m. The content of eupatilin showed significant variability across all altitude ranges. In the comparison of the other seven ingredients measured in both leaves and stems, the most significant disparity was observed in the below 500 m range, with differences becoming less pronounced at higher altitudes. At the same time, we analyzed the correlation between the contents of eight flavonoids and phenolic acids in leaves and stems (**Figure 7B**). The *R^2^* values for neochlorogenic acid and cryptochlorogenic acid were 0.616 and 0.673, respectively, indicating a significant correlation between them (*P*<0.05). No statistically significant correlations were found for chlorogenic acid, isochlorogenic acid B, isochlorogenic acid A, isochlorogenic acid C, jaceosidin and eupatilin.

Three principal components (PC1, PC2, PC3) were extracted from the principal component analysis of the main medicinal ingredients in different organs of *A. argyi* across various altitudes, yielding a cumulative interpretation rate of 84.620%. This rate effectively encapsulates the information regarding the main medicinal ingredients in different organs of *A. argyi* at varying altitudes (**Table 7**). The variance contribution rate of PC1 was 55.700%, characterized by substantial absolute values of the eigenvectors for the contents of eight flavonoids and phenolic acids in leaves, as along with neochlorogenic acid and cryptochlorogenic acid in stems. Therefore, PC1 mainly reflects the contents of the primary medicinal components in the leaves. PC2 accounted for a variance contribution rate of 21.543%, highlighting larger absolute values of the eigenvectors for the contents of chlorogenic acid, isochlorogenic acid B, isochlorogenic acid A and isochlorogenic acid C in stems. Therefore, PC2 primarily reflects the information related to the phenolic acids in the stems. PC3 contributed a variance rate of 7.377%, with larger absolute values of the eigenvectors for the contents of jaceosidin and eupatilin in stems, indicating that PC3 predominantly reflects the content of the flavonoids in the stems. Together, PC2 and PC3 provide insights into the main medicinal components in the stems, with a cumulative contribution rate of 28.920%.

**Table 7:** Principal component analysis of main medicinal ingredients in different organs at different altitudes.

Organs	Indexes	PC1	PC2	PC3
Leaves	Neochlorogenic acid	0.987	-0.03	-0.042
	Chlorogenic acid	0.971	-0.113	-0.084
	Isochlorogenic acid B	0.977	-0.129	-0.017
	Isochlorogenic acid C	0.973	-0.019	-0.12
	Jaceosidin	0.786	-0.259	-0.171
	Eupatilin	-0.735	0.489	0.067
	Cryptochlorogenic acid	0.954	0.024	-0.009
	Isochlorogenic acid A	0.966	0.012	-0.138
Stems	Neochlorogenic acid	0.790	0.391	0.375
	Chlorogenic acid	0.521	0.779	0.054
	Isochlorogenic acid B	-0.139	0.930	-0.086
	Isochlorogenic acid C	0.498	0.664	0.211
	Jaceosidin	-0.361	-0.262	0.659
	Eupatilin	0.369	-0.289	0.668
	Cryptochlorogenic acid	0.815	-0.122	0.163
	Isochlorogenic acid A	0.008	0.936	0.050
Initial eigenvalue		8.912	3.447	1.180
Contribution rate/%		55.700	21.543	7.377
Accumulative contribution rate/%		55.700	77.243	84.620

PC1, PC2 and PC3 represent principal component 1, principal component 2 and principal component 3, respectively.

## 4 Discussion

### 4.1 Differences in yield and medicinal quality of wild populations of *A. argyi* at different altitudes

Altitude is a crucial environmental factor that profoundly influences the spatial distribution, growth, and development of plants. As altitude increases, temperature decreases, diurnal temperature variations become more pronounced, and light intensity increases (Feng et al., 2022; Yao et al., 2023). Light plays a vital role in the development, productivity and quality of crops (Su et al., 2024). In this study, individual yield values were notabily higher in four regions (<500 m, 500-600 m, 700-800 m, and 900-1000 m), while lower values were observed in the remaining three regions. TRP was higher in the <500 m and 500-600 m ranges, where habitats were sunnier and drier. Although the TRP in the 700-800 m and 900-1000 m ranges was lower, reduced population density increased light transmittance. Moreover, *A. argyi* in the 700-800 m and 900-1000 m ranges showed relatively tall plant heights, establishing a significant positive correlation between plant height and individual yield. Furthermore, the increased altitude resulted in stronger light intensity, which facilitated the optimal utilization of light energy by *A. argyi*. As a heliophytes, *A. argyi* adapts to the variation in habitats to maximize its light energy for absorption.

Altitude can also significantly influence the synthesis of medicinal ingredients. The response of different medicinal plants to altitude varies. For instance, the root biological activity of *Paeonia veitchii* growing at high altitudes was higher, which was associated with a lower annual mean temperature (Yuan et al., 2020). Additionally, higher altitude may enhance the biosynthesis of flavonoids in *Ginkgo biloba* leaves, mainly due to increased light exposure associated with altitude (Zou et al., 2019). Volatile ingredients are significantly affected by sunshine duration and temperature, with the effect of low temperatures being more pronounced than that of high temperatures (Wang et al., 2018). Adequate lighting can enhance the output rate of moxa, as well as increase the levels of flavonoids and phenolic acids (Li et al., 2023). In this study, the medicinal quality was highest at altitudes below 500 m, while altitudes between 500-600 m, 600-700 m, and 800-900 m showed similar qualities, with significant differences among the other four altitude intervals. The populations surveyed at high altitudes experienced low TRP in their habitats and received relatively few hours of adequate sunshine. Although the total volatile oil content and eupatilin levels reached higher values at 900-1000 m, the output rate of moxa and the content of jaceosidin, eucalyptus oleoresin, borneol, and six phenolic acids were lower in the wild populations of *A. argyi* located at these higher altitudes. Moreover, the accumulation patterns of eupatilin were found to be fundamentally opposite to those of jaceosidin and the six phenolic acids, suggesting that the synthesis of flavonoids is more susceptible to interference compared to other phenylpropanoids (phenolic acids) in response to changes in altitude.

### 4.2 Ecological factors influence the yield and medicinal quality of *A. argyi.*

*A. argyi* shows strong community ecological niche competitiveness and a reduced presence of weeds in its natural habitat, facilitating the establishment of a dominant population (Chen et al., 2022). This dominance can effectively minimize factors that interfere with yield and medicinal quality. Planting density in cultivation is a fundamental factor for achieving a balance between population and individual development. Thinning practices can effectively reduce leaf drop rates and increase the number of effective leaves; however, they also lead to a decrease in the content of eucalyptus oleoresin. On the other hand, dense planting can significantly increase yield, output rate of moxa, and concentrations of phenolic acids, such as chlorogenic acid in *A. argyi* (Ma et al., 2020). In this study, a positive correlation was observed between population density and the medicinal quality of *A. argyi* (*P*<0.05). While there was a significant positive correlation between population density and medicinal quality, no considerable relationship was noted with yield. Population density emerged as the only significant factor among community characteristics factors, indicating its key role in affecting the medicinal quality of *A. argyi*. Appropriate dense planting practices prove beneficial for improving the medicinal quality of *A. argyi*, aligning with the conclusions drawn from cultivation. The lack of significance in the relationship between density and yield may be attributed to the uneven distribution of *A. argyi* in wild communities. Understanding the key roles of nutrient elements in soil–plant systems is essential for the production of herbal medicine and sustainable development (Qian et al., 2024). The yield of *A. argyi* leaves and moxa initially increases with N fertilizer application, but subsequently decreases at higher N levels. Under conditions of N deficiency or low N levels, the synthesis of borneol is hindered. However, N application enhances the photosynthetic rate and facilitates the accumulation of carbon-based secondary metabolites such as phenolic acids and flavonoids (Ma et al., 2021a). The synergistic effects of P and N enhance photosynthesis and promote dry matter accumulation (Chen et al., 2021). However, the P content in *A. argyi* leaves negatively impacts both yield and total volatile oil content (Chen et al., 2022a). The key enzyme genes involved in the synthesis of flavonoids and phenolic acids are upregulated in the absence of P (Wang et al., 2023b). Potassium is linked to photosynthesis, and can promote the accumulation of Ca and increased gibberellin levels in *A. argyi*, which in turn encourages the growth of non-glandular hair and increases the output rate of moxa (Chen et al., 2022b).

In this study, soil TN content showed a significant positive correlation with yield while negatively correlating with medicinal quality. TP content was significantly negatively correlated with both yield and medicinal quality. This suggests that soil TN and TP contents are key driving factors affecting yield and medicinal quality. Altitude significantly influences soil TN content, and a synergistic relationship between TN content and LNA suggests that altitude may alter the N absorption of *A. argyi*. N/P and TP, C/P and SPC, SOC and LPC showed antagonistic relationship, which might be related to soil mineralization. The contents of N and P influence each other, with lower C/P values promoting increased available P in the soil (Liu et al., 2018), thereby affecting the uptake and utilization of P by *A. argyi*. In leaves and stems, the contents of N and P coexist synergistically, while the accumulation of N and P, K in leaves also exhibit a synergistic relationship. The combined effects of N, P, and K improves photosynthesis and the number of non-glandular hairs, resulting in increased individual yield. Excessive P can negatively affect *A. argyi*, especially affecting the contents of N, K and Ca, leading to nutritional imbalances and a reduction in output rate of moxa (Chen et al., 2021; Wang et al., 2023b). Consequently, both individual yield and medicinal quality of *A. argyi* decline. The altitude range associated with the highest contents of P in plant organs and soil TP was consistent in the study, yet both individual yield and medicinal quality were low. In conclusion, changes in altitude can affect soil mineralization, altering the availability of N and P that A. argyi can directly utilize. This, in turn, influences the absorption of mineral elements, impacting both the individual yield and medicinal quality of *A. argyi*.

### 4.3 Difference of main medicinal ingredients in different organs of *A. argyi*

Different organs of medicinal plants contain similar medicinal ingredients, but their distribution and accumulation characteristics can vary significantly. In *Sapindus mukorossi*, the distribution of total saponins and total flavonoids among different organs, particularly in leaves, exhibited notable differences. An indirect competitive relationship exists between the accumulation of saponins and flavonoids (Xu et al., 2021). In *Lycium barbarum*, the leaves are referred to as Tianjingcao, the flowers as Changshenghua, the root bark as Cortex lycii, and the fruit as Lycium Chinense. The roots, stems, and leaves of *Lycium barbarum* work in coordination to produce high-quality fruits (Ma et al., 2024). The roots serve as the primary medicinal part of *Aconitum carmichaelii* Debx. The lateral root processing product is known as radix aconiti lateralis preparate, while the mother root processing product is simply *Aconitum carmichaelii*. The stems and leaves of *Aconitum carmichaelii* Debx. are rich in aporphine alkaloids and alcohol ammonia diterpenoid alkaloids, which possess potential anti-tumor properties and therapeutic effects for rheumatic diseases, along with low levels of toxic ingredients (Zhou et al., 2024). The antioxidant capacity of lotus seeds is significantly higher than that of other parts of the ancient Chinese lotus (Wang et al., 2023). In this study, the main medicinal ingredients in the organs of *A. argyi* were found to be sensitive to variations in altitudes. The contents of the main medicinal ingredients in the leaves of *A. argyi* collected from altitudes below 500 m was much greater than that in the stems. Therefore, the primary focus should be on utilizing and developing the leaves. At high altitudes, the differences in the content of main medicinal ingredients between leaves and stems is minimal, particularly regarding chlorogenic acid and isochlorogenic acid B, where the phenomenon of stem content surpassing leaf content occurred. This suggests that the medicinal value of the stems could be explored based on altitude range and associated medicinal ingredients. A significant correlation and synergistic effect were observed between the contents of neochlorogenic acid and cryptochlorogenic acid in both leaves and stems, while the other six ingredients did not show close relationships. Principal component analysis showed that the contents of eight flavonoids and phenolic acids were higher in the leaves, reflecting their medicinal quality. The presence of neochlorogenic acid and cryptochlorogenic acid in the stems was also part of the first principal component, indicating a significant correlation with corresponding indices in the leaves. Therefore, the evaluation of the medicinal quality of *A. argyi* should primarily focus on its leaves, while also taking into the potential medicinal value of its stems.

## 5 Conclusions

The yield and medicinal quality of *A. argyi* vary significantly due to altitude and ecological factors, such as community characteristics and soil chemical properties. In the study area, both the yield and medicinal quality of *A. argyi* were found to be higher at altitudes below 500 m. Key factors influencing these attributes include population density, soil TN content, and TP content. Soil mineralization characteristics differ markedly across different altitudes, which directly affects the absorption of mineral elements by *A. argyi*. A significant positive correlation was observed between the accumulation of N, P, and K in leaves and yield. Conversely, the P content in leaves and stems showed a significant negative correlation with yield and medicinal quality, respectively. In practice, it is advisable to select cultivation areas below 500 m in altitude with near neutral soil for *A. argyi*. In addition, it is important to control planting density and strictly regulate the application of N, P, and K fertilizers, especially N and P. Compared to the stems, the leaves of *A. argyi* leaves more accurately reflect its medicinal quality. However, the stems also possess medicinal value. Therefore, it is recommended to explore the development of stems based on altitude and their medicinal ingredients.

## Data Availability

The original contributions presented in the study are included in the article/supplementary material. Further inquiries can be directed to the corresponding author/s.
